# An Eye Tracking and Event-Related Potentials Study With Visual Stimuli for Adolescents Emotional Issues

**DOI:** 10.3389/fpsyt.2022.933793

**Published:** 2022-06-30

**Authors:** Quan Wang, Xiaojie Wei, Ruochen Dang, Feiyu Zhu, Shaokang Yin, Bingliang Hu

**Affiliations:** ^1^Key Laboratory of Spectral Imaging Technology, Xi’an Institute of Optics and Precision Mechanics of the Chinese Academy of Sciences, Xi’an, China; ^2^University of Chinese Academy of Sciences, Beijing, China; ^3^Key Laboratory of Biomedical Spectroscopy of Xi’an, Xi’an Institute of Optics and Precision Mechanics of the Chinese Academy of Sciences, Xi’an, China

**Keywords:** adolescent, depression, mania, eye-tracking, ERP, N1, emotion

## Abstract

**Background:**

Psychological issues are common among adolescents, which have a significant impact on their growth and development. However, the underlying neural mechanisms of viewing visual stimuli in adolescents are poorly understood.

**Materials and Methods:**

This study applied the Chinese version of the DSM-V self-assessment scales to evaluate 73 adolescents’ psychological characteristics for depressive and manic emotional issues. Combined with eye-tracking and event-related potential (ERP), we explored the characteristics of their visual attention and neural processing mechanisms while freely viewing positive, dysphoric, threatening and neutral visual stimuli.

**Results:**

Compared to controls, adolescents with depressive emotional tendencies showed more concentrated looking behavior with fixation distribution index than the controls, while adolescents with manic emotional tendencies showed no such trait. ERP data revealed individuals with depressive tendencies showed lower arousal levels toward emotional stimuli in the early stage of cognitive processing (N1 amplitude decreased) and with prolonged reaction time (N1 latency increased) than the control group. We found no significant difference between the manic group and the control group. Furthermore, the depression severity scores of the individuals with depressive tendencies were negatively correlated with the total fixation time toward positive stimuli, were negatively correlated with the fixation distribution index toward threatening stimuli, and were positively correlated with the mean N1 amplitudes while viewing dysphoric stimuli. Also, for the individuals with depressive tendencies, there was a positive correlation between the mean N1 amplitudes and the fixation time on the area of interest (AOI) while viewing dysphoric stimuli. For the individuals with manic tendencies, the manic severity scores of the individuals with manic tendencies were positively correlated with the total fixation time toward the positive stimuli. However, no significant correlations were found between the manic severity scores and N1 amplitudes, and between N1 amplitudes and eye-tracking output variables.

**Conclusion:**

This study proposes the application of eye-tracking and ERP to provide better biological evidence to alter the neural processing of emotional stimuli for adolescents with emotional issues.

## Introduction

Adolescence is a unique lifespan period when individuals frequently encounter major life transitions and their minds are not yet mature. At the same time, they were also burdened by academic pressure and interpersonal pressure, which can easily breed emotional issues. Emotional issues are highly prevalent and debilitating performances characterized by social fears, worries, depression, and mania in adolescence (1–3). Also, emotional issues significantly affect the growth and development of adolescents. Data show that about 2–8% of adolescents are affected by emotional issues, and the number is moving in an increasing trend year by year (4). At the same time, the early detection of emotional issues in adolescents encounters many challenges currently. Firstly, the information from the adolescents is subjective. Because most adolescents have no scientific understanding of emotional issues, they often feel ashamed and refuse to talk to doctors when visiting a doctor, which affects the doctor’s diagnosis and treatment. Secondly, there are no quantitative diagnostic criteria. Usually, with the assistance of a questionnaire, the final diagnosis results rely on the clinical experience of the doctor, which has a strong subjective influence (5). Thirdly, teenagers are in a rebellious period, and their attitude may not cooperate and follow precisely the doctor’s diagnosis procedures, which increases the difficulty of the doctor’s diagnosis. Therefore, it is of great importance to find an objective and quantitative method to promote the detection of emotional issues.

Negative childhood experiences, especially emotional issues, played significant roles in developing specific biases in processing information (6). As early as 1976, American Psychologist Beck (7) proposed that affective disorders arise from negative cognitive schemas. Individuals with emotional disorders showed more negative cognitive biases in information processing, including attention, memory, interpretation, and many other aspects, which would aggravate their negative emotional state. Many information-processing studies have shown that individuals with depressive tendencies tended to choose the one consistent with their negative cognitive schemas when external processing information. That is, there was a negative cognitive processing bias. Moreover, mania or hypomania (cardinal symptoms of bipolar disorder) was associated with dysregulated emotional responses (8–10).

Recent research has made significant progress elucidating an association between emotional issues and biased attention. Eizenman et al. (11) used eye-tracking technology to find that depressed individuals spent significantly more time looking at negative pictures than non-depressed controls. Another study examined college student participants of mania had a positive correlation with an attentional bias toward happy faces (12). Despite these initial results, it remains inconclusive about the patterns of attentional biases in adolescents of emotional issues proneness as well as prior to the formal onset of emotional disorder.

Previous studies have often used primary eye movement indices to describe different emotional problems, such as total fixation duration, total fixation duration of interest areas, and fixation distribution index (13, 14). Liu et al. (15) considered eye-movement indices of the total fixation duration in the free-viewing task. Results found manic patients had a shorter total fixation duration on sad images and neutral images than healthy controls which reflected an avoidance of sad expressions. However, there was no significant difference in happy images between the manic and control group. Kim et al. (16) calculated the total fixation duration of interest areas (eyes, nose, mouth). Results found that children with bipolar disorder spent less time looking at the eyes than the healthy controls, regardless of the facial emotion (anger, fear, sadness, happiness, neutral). Philip et.al (13) assessed the fixation distribution using the dispersion coefficient of the eye movement index and found that the spatial distribution of cannabis-induced psychosis was more concentrated than that of first-episode schizophrenia and healthy controls.

To delineate the response of the cerebral cortex that is directly related to stimulus processing in the free-viewing picture paradigm, event-related potentials (ERP) is an effective technique to study cognitive information processes related to real-time brain activity in the range of milliseconds. Combined with self-report measures, ERPs have been shown to predict depression outcomes (17–19) independently. ERP components have been confirmed to be related to specific aspects of information processing.

The N1 is a specific ERP component that has been systematically related to the processing of emotional stimuli (20). The early negative component N1 is sensitive to physical stimuli and is associated with attentional processing (21), and N1 peaks at around 130–200 milliseconds after stimulus triggers, mainly at the occipitotemporal electrodes (22–24). Some studies have discovered that N1 amplitude is associated with emotional expression, while others have not found this sensitivity to emotion (25).

The N1 component is commonly associated with emotional issues. Reductions of the N1 components have been consistently reported in depressed individuals (26, 27). JA Coffman et al. (26) adopted an “auditory oddball” paradigm. Results found depressed individuals who had a mean age of 50 years had lower N1 amplitude and longer N1 latency than controls before therapy treatments. But after the treatment, they have no significant difference. Urretavizcaya M et al. (27) also adopted an “oddball paradigm” in the auditory modality. Results found depressed adult patients showed a significantly higher latency in N1, N2, and P3 than healthy controls. O’Donnell BF et al. (28) examined the N100 component of ERPs that were elicited during an auditory discrimination task in which participants pressed keys to unusual 1,500 Hz tones interspersed in a series of 1,000 Hz tones. They reported that there was no significant difference between bipolar disorder patients and healthy control in the N1 component. However, less research has been researched on the N1 component for visual stimuli in adolescents with emotional issues.

In order to explore potential systemic changes in eye-tracking and ERP components during relatively long stimulus presentations, we adopted positive, dysphoric, threatening, and neutral stimuli and analyzed the eye-tracking gaze characteristics and EEG characteristics of adolescents with different emotional issues. To further explore whether eye-tracking measures and ERP amplitudes elicited by emotional stimuli prospectively predict the severity of emotional issues, we investigated the relationship between eye-tracking measures, ERP components, and scale scores of self-report measures, expecting early detection and early prevention of emotional issues. Based on the mood-congruent attentional bias posited by cognitive patterns of depression raised by Beck et al. (7) and previous research, we hypothesized that adolescents with depressive tendencies pay more attention to dysphoric stimuli and less attention to positive stimuli. According to the study by June Gruber et al. (12) who recruited Emerging Adulthood (ages 18–25) of hypomania proneness and found that hypomania was positively associated with happy faces. Thus we hypothesized adolescents with manic tendencies are positively associated with positive stimuli. The research on the N1 components for depressive and manic tendencies has not yet formed a consistent conclusion, therefore further research is needed in this experiment.

## Materials and Methods

### Participants

A total of seventy-three male adolescents (age range 14–17 years, Mean age = 16.14, SD = 0.65) took part in the present study and signed the written informed consent form. All individuals were recruited through advertisements and by word of mouth from local high schools. Inclusion criteria for the current study were as follows: (1) Right-handed; (2) Age 11–17 years old; (3) No medication-taking that affects nervous system function within 2 weeks, no alcohol dependence or other drug dependence; (4) Normal vision or corrected vision, no color blindness or other ophthalmic diseases; and (5) No facial expression recognition disorder and normal language communication. Exclusion criteria for the current study were as follows: (1) Individuals with psychotic symptoms; (2) developmental delay; (3) learning disabilities; and (4) recurrent or chronic pain.

DSM-V online self-assessment scale was used to identify participants who met the criteria for inclusion into one of three groups: (a) individuals with depressive tendencies who met the criteria of the Chinese version of DSM-V level 2-Depression-Child Age 11–17 scale; (b) individuals with manic tendencies who meet the criteria of Altman Self-Rating Mania Scale (ASRM); and (c) healthy controls who had no psychiatric or neurological disorders. In the eye-tracking study, there were 16 individuals with depressive tendencies (mean age 16.13 years), 19 individuals with manic tendencies (mean age 16.05 years), and 20 individuals in the healthy control group (mean age 16.25 years). Due to the previous acquisition, the EEG had not been set, and just only eye-tracking data were collected. In the synchronization of the EEG and eye-tracking study, there were 10 individuals with depressive tendencies (mean age 16.33 years), 12 individuals with manic tendencies (mean age 15.67 years), and 16 individuals in the healthy control group (mean age 16.00 years). Group characteristics such as age and scale scores are listed in Table 1 for all participant groups.

**TABLE 1:** Group characteristics (Mean ± SD).

A study population of eye-tracking			
Characteristic	Control (*n* = 20)	Depression (*n* = 16)	Manic (*n* = 19)
Age	16.25 0.44	16.13 0.62	16.05 ± 0.83
PROMIS emotional distress-depression-pediatric item bank	22.30 5.30	35.56 3.50	23.00 5.67
Altman self-rating mania scale	3.551.63	5.38 2.36	9.112.85
**A study population of EEG and Eye-tracking**			
Characteristic	Control (*n* = 16)	Depression (*n* = 10)	Manic (*n* = 12)
Age	16.00 0.52	16.330.52	15.670.78
PROMIS emotional distress-depression-pediatric item bank	23.69 4.42	35.80 4.19	22.42 6.26
Altman self-rating mania scale	3.31 ± 2.06	6.00 1.91	9.75 3.17

*For all tests, mean scores and standard deviations (SD) are reported.*

### Procedure

The whole experiment was carried out in a quiet and stable electromagnetic environment with constant illumination. After entering the experimental room, participants read the study instructions, signed the consent form, and then were surveyed by the online scale of Diagnostic and Statistical Manual of Mental Disorders (DSM-V), including the DSM-V level 2-Depression-Child Age 11–17 and the ASRM. Then the main tester measured the head circumference, selected a suitable electrode cap, placed and adjusted the electrode location with the Cz electrode as a reference (located in the center of the EEG cap), and injected potassium chloride solution into the sponge of the electrode to reduce the electrode impedance until the impedance of the electrode dropped below 50 kΩ (29, 30). Subjects sat 60 cm in front of the eye-tracker system. Camera adjustments were made to best capture the participant’s eye, and a 9-point calibration was complete to confirm that the eye tracker was recording a line of visual gaze within 1.25° of visual angle. After successful calibration and validation of the eye-tracking position, experimental stimuli were presented with the same pseudo-randomized sequence of images. The process took approximately 10 min to complete. Finally, small prizes such as pens and notebooks were distributed to the subjects.

### Assessments

#### Depressive Symptom Severity

Participants completed the Chinese version of DSM-V level 2-Depression-Child Age 11–17, On 14 items rated on a five-point scale, from “never” to “always,” a total score varying from 14 to 70. This measure has demonstrated that higher scores represented higher levels of depression. At the same time, the measure also reflected the extent to which they suffered from depressive symptoms within the past 2 weeks. The depression scale scores were greater than 31 as the selection criteria for the depressed group.

#### Manic Symptom Severity

The ASRM is a short, 5-item self-assessment questionnaire that can help assess the severity of manic or hypomanic, with a total score ranging from 0 to 20 (31). Although the scale is concise, it is compatible with the CARS-M, YMRS, and DSM-IV diagnostic criteria and can still be used effectively as a screening and diagnostic tool. Participants were instructed to endorse only one of the five statements from each item, rated in increasing severity from 0 (not present) to 4 (present in severe degree), that best described their mood or behavior during the past week. The mania scale scores were greater than five as the selection criteria for the manic group.

### Stimulus and Task

#### Materials

**FIGURE 1:**
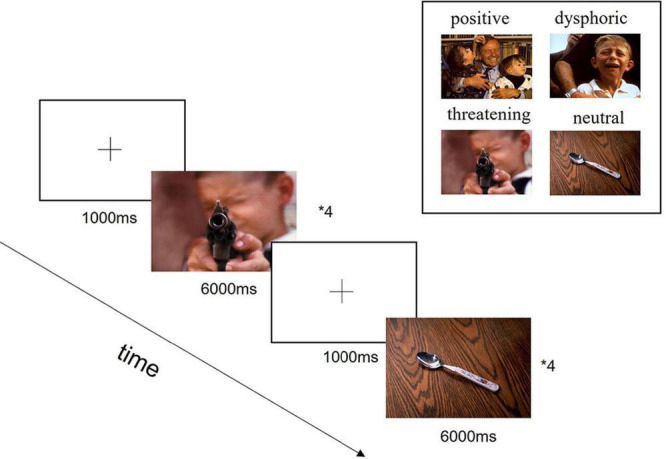
Experimental design diagram. In the beginning, each trial was presented a fixation cross in the center of the screen for 1,000 ms, followed by the presentation of stimuli for 6,000 ms. Each block contained four trials with specific emotions and four trials with neutral images. The aim of the neutral images added was to confuse the experimental purpose and reduce subjects to guess the experimental intention, which would affect the collected data. A total of 9 blocks were presented in a pseudo-randomized order.

In this study, a total of 72 images were selected from the International Affective Picture System (IAPS), including 12 positive stimuli, 12 dysphoric stimuli, 12 threatening stimuli, and 36 neutral stimuli. The images from the IAPS provide standardized emotional stimuli and have been used widely in neuropathology research. The size and resolution of the images used in this experiment kept the same of 1024 × 768 pixels, as shown in Figure 1.

The 72 images that we used are from IAPS image set of Kellough et al. (32). Not only have these images been systematically evaluated for arousal and valence, but also the arousal and valence are consistent. In addition, the IAPS images are widely used in human emotion research worldwide and make an important contribution to the evolving database in emotional research (33).

#### Apparatus

In this study, eye movements were recorded from the left eye with our independently developed EyeCatch Bar desktop eye-tracker system, collecting gaze data at a 41 Hz (coordinates were sampled every 24.4 ms), with an accuracy of 1.25°. Participants’ eyes were kept at a distance of 60 cm apart from the eye tracker.

Before the experiment, each subject underwent eye movement calibration. Nine dots had to be fixed one after the other, in the center of the screen, and the other shifted to the top, bottom, left, right, the upper left, the upper right, the lower left, the lower right, respectively. Last, back to the center. Afterward, the same order was followed for verification. The recorded eye-tracking data was analyzed using custom-made analysis code in Python.

We recorded continuous EEG data from 64 channels (HydroCel Geodesic Sensor Net, Electrical Geodesics, Inc., Eugene, OR, United States) with Net Station EEG Software. All electrodes were physically referenced to Cz (fixed by the EGI system), and then the mean value of the left and right mastoids was used for offline re-reference. The impedance of all electrodes was kept below 50 kΩ during data acquisition. The EEG was amplified with a bandpass of 0.1–70 Hz (half-power cutoff) and digitized online at 250 Hz.

The collected EEG data were preprocessed using EEGLAB environment, a tool library for processing EEG data in MATLAB. In this experiment, all EEG signals were filtered using a 0.1 Hz high-pass filter and a 30 Hz low-pass filter to reduce noise. For the data after ICA, we used the Adjust plugin in EEGLAB to discard artifacts due to eye movement and muscle activity. The data were then segmented relative to stimulus onset (−200 to 800 ms), and the baseline preceding the stimulus (−200 to 0 ms) was subtracted. Trials with excessive movements or eye blinks (voltage exceeding 100 μV) were automatically rejected. We computed the grand-averaged ERP waveforms for different groups.

### Statistical Analysis

#### Eye-Tracking Analysis

Three primary eye-tracking indices of the free viewing task were performed: (a) total fixation time (the sum of durations from all fixations on a specific stimulus); (b) fixation time in the area of interest (AOI) (the sum of durations from the fixation on the most representative emotional area); and (c) fixation distribution index (13, 14). To analyze the patterns of attention distribution or clustering of fixations in adolescents with emotional issues when looking at different emotional images, the fixation distribution index was assessed. The value of alpha denoted the degree of fixation distributions. Higher alpha denotes more dispersed distributions (13).

We used descriptive and graphical methods to test whether the data met a normal distribution. Then eye-movement measures were analyzed in 3 Groups (control, depressed, manic) × 4 Emotional Stimuli (positive, dysphoric, threatening, neutral) analysis of variance (ANOVA) in which Groups were a between-subject factor and Emotional Stimuli were a within-subject factor. Significant interactions were analyzed using simple effects models. The significance level was set at 0.05.

#### Event-Related Potential Analysis

Statistical analysis was performed using a semi-automatic peak picking program with ERPLAB tools to calculate amplitude and latency. The time windows locked in each peak were selected. ERP component N1 was found in different stages of attentional processing. Based on the scalp topographic distribution of mean ERP activity calculated in the previous studies (34), a set of electrodes were used for statistical analysis of ERP components. The following nine electrode sites (F3, Fz, F4, C3, Cz, C4, P3, Pz, P4) were selected as in previous literature (35–37) and classified into three regions: frontal (F3, Fz, F4), central (C3, Cz, C4), parietal and (P3, Pz, P4), where N1 component (120–190 ms after stimuli onset) had maximal amplitude for statistical analysis. 3 Groups (control, depressed, manic) × 4 Emotional Stimuli (positive, dysphoric, threatening, neutral) × 3 Electrode Regions (Frontal, Central, Parietal) design of ANOVA statistical analysis was designed for the N1 amplitude and latency. The effect sizes in ANOVAs were shown as partial eta squared (η^2^). The degrees of freedom were adjusted using Greenhouse-Geisser corrections when appropriate. We performed subsequent analyses with IBM SPSS Statistics Version 22.0.

#### Correlation Analysis

In addition, relationships between the DSM-V scale scores (DSM-V level 2-Depression-Child Age 11–17 Scale and the ASRM) and eye-tracking measures (total fixation time, fixation time in the AOI, fixation distribution) or ERP components (N1 amplitudes) were calculated by the Spearman correlation analysis. Similarly, the correlation between eye-tracking measures and N1 amplitudes was also analyzed by the Spearman correlation analysis.

## Results

### Group Differences in Eye-Tracking Data

#### Total Fixation Time

**FIGURE 2:**
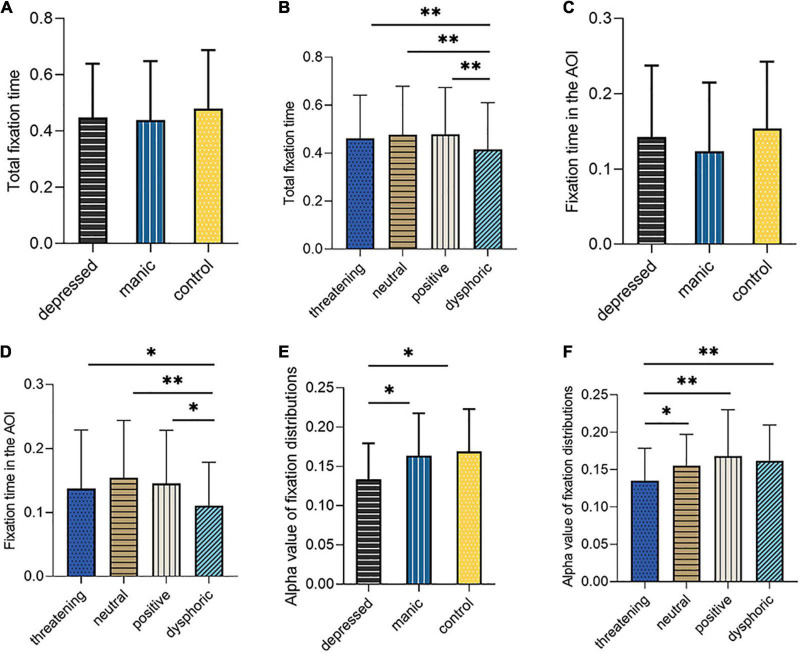
The schematic illustration in **(A)** shows total fixation time according to different groups, in **(B)** shows total fixation time according to different stimulus type, in **(C)** shows AOI fixation time according to different groups, in **(D)** shows AOI fixation time according to different stimulus type, in **(E)** shows the alpha value of fixation distribution according to different groups, in **(F)** shows the alpha value of fixation distribution according to different stimulus type. ^∗^Significant difference at *p* < .05. ^∗∗^Significant difference at *p* < 0.01.

A repeated-measure ANOVA on total fixation time while viewing picture with Greenhouse-Geisser correction, with stimulus type (positive, dysphoric, threatening, neutral) group (control, depressed, manic) yielded a not significant interaction [*F*_(6,102)_ = 1.907, *P* = 0.103, η^2^ = 0.068]. The main effect of subject groups [*F*_(2,52)_ = 0.232, *P* = 0794, η^2^ = 0.037] was not significant (Figure 2A). Only the main effect of stimulus type was statistically significant [*F*_(3,50)_ = 7.488, *P* < 0.001, η^2^ = 0.126]. *Post-hoc* analyses indicated that adolescents had longer total fixation time while looking at the neutral stimuli (*M* = 0.474, *P* < 0.001), positive stimuli (*M* = 0.482, *P* < 0.001) and threatening stimuli (*M* = 0.448, *P* = 0.041) rather than dysphoric stimuli (*M* = 0.420) (Figure 2B).

#### Area of Interest Fixation Time

A repeated-measure ANOVA on AOI fixation time with Mauchly’s test of sphericity, with stimulus type (positive, dysphoric, threatening, neutral) × group (control, depressed, manic) yielded a not significant interaction [*F*_(6,102)_ = 1.357, *P* = 0.235, η^2^ = 0.050]. The main effect of subject groups (*F*_(2,52)_ = 0.740, *P* = 0.482, η^2^ = 0.028) was not significant (Figure 2C). Only the main effect of stimulus type was statistically significant [*F*_(3,50)_ = 8.725, *P* < 0.000, η2 = 0.144]. *Post-hoc* analyses indicated that adolescents had longer AOI fixation time while looking at the neutral stimuli (*M* = 0.162, *P* < 0.001), positive stimuli (*M* = 0.147, P = 0.001) and threatening stimuli (*M* = 0.140, *P* = 0.029) than dysphoric stimuli (*M* = 0.112) (Figure 2D).

#### Fixation Distribution Index

A repeated-measure ANOVA on alpha value of dispersion coefficient with Mauchly’s test of sphericity, with stimulus type (positive, dysphoric, threatening, neutral) group (control, depressed, manic) was performed. A main effect was found for the subject groups [*F*_(2,52)_ = 4.337, *P* = 0.018, η^2^ = 0.143] (Figure 2E), indicating that the depressed group (*M* = 0.134) exhibited lower alpha value than the control group (*M* = 0.169, *P* = 0.007) and the manic group (*M* = 0.163, *P* = 0.025). In addition, the analysis of alpha value revealed a significant main effect for stimulus type [*F*_(3,50)_ = 10.817, *P* < 0.000, η^2^ = 0.172]. Across different stimulus types, the dispersion coefficient of fixation while viewing the positive stimuli (*M* = 0.173, *P* < 0.001), neutral stimuli (*M* = 0.157, *P* < 0.001) and dysphoric stimuli (*M* = 0.159, *P* = 0001) were higher than threatening stimuli (*M* = 0.132). The alpha results are depicted in (Figure 2F). No group stimulus type interaction was detected [*F*_(6,102)_ = 1.756, *P* = 0.112, η^2^ = 0.063].

### Group Differences of Event-Related Potential Data

#### N1 Component (120–190 msec).

**FIGURE 3:**
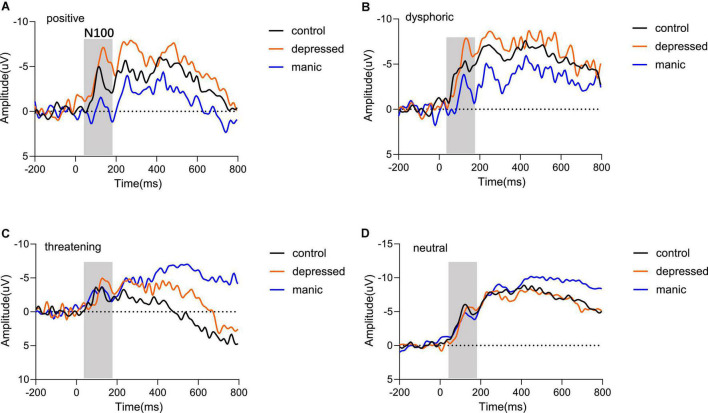
Grand averaged waveforms of mean electrodes for different emotional stimuli. The comparison of the three groups regarding N100 components in **(A)** positive stimulus type, **(B)** dysphoric stimulus type, **(C)** threatening stimulus type, and **(D)** neutral stimulus type. Odd numbers represent electrode locations on the left hemisphere, whereas even numbers represent those on the right hemisphere.

The N1 component of this time window are shown in Figure 3. The three-way repeated measures ANOVA on N1 component, with 3 Groups (control, depressed, manic) × 4 Emotional Stimuli (positive, dysphoric, threatening, and neutral) × 3 Electrode Regions (Frontal, Central, Parietal), showed a main effect of group [*F*_(2,35)_ = 3.410, *P* = 0.044, η^2^ = 0.163], the depressed group (*M* = −7.477) elicited a significantly lower N1 amplitude than the control group (*M* = −5.025, *P* = 0.002). In addition, there was no significant difference between the manic group (*M* = −5.162) and the control group (*M* = −5.025, *P* = 0.886) (Figures 3A–D). A significant effect of electrode regions [*F*_(2,34)_ = 146.396, *P* < 0.000, η^2^ = 0.807], indicated that N1 amplitudes were generally larger at parietal (*M* = −2.580) than the central sites (*M* = −7.024, *P* < 0.000), and the N1 amplitudes at parietal sites were also larger than that at the frontal sites (*M* = −8.061, *P* < 0.000). The main effect of stimulus type was not statistically significant [*F*_(3,33)_ = 0.682, *P* = 0.541, η^2^ = 0.019]. Moreover, no interaction effects were found over N1 amplitude.

We also analyzed N1 latency and found a significant main effect of the group [*F*_(2_,_35)_ = 4.316, *P* = 0.021, η^2^ = 0.198] and stimulus type [*F*_(3_,_33)_ = 3.026, *P* = 0.043, η^2^ = 0.216], but the electrode regions was not significant [*F*_(2_,_34)_ = 0.180, *P* = 0.786, η^2^ = 0.005]. Moreover, no interaction effects were found over N1 latency. *Post-hoc* analyses found that the N1 latency of the depressed group (*M* = 129.567) was significantly greater than the control group (*M* = 126.802, *P* = 0.010). However, there was no significant difference between the manic (*M* = 126.495, *P* = 0.767) and the control group. For the type of stimuli, neutral stimuli (*M* = 128.866) (Figure 3D) have longer latency than the stimuli with emotional content. The neutral stimuli latency was significantly longer than the latency toward positive stimuli (*M* = 127.225, *P* = 0.032) (Figure 3A) and dysphoric stimuli (*M* = 126.924, *P* = 0.009) (Figure 3B), and was marginally longer than the threatening stimuli (*M* = 127.470, *P* = 0.092) (Figure 3C).

### Association Analysis

#### Associations Between Depression Scale Scores and Eye-Tracking Measures Among Participants With Depression

**FIGURE 4:**
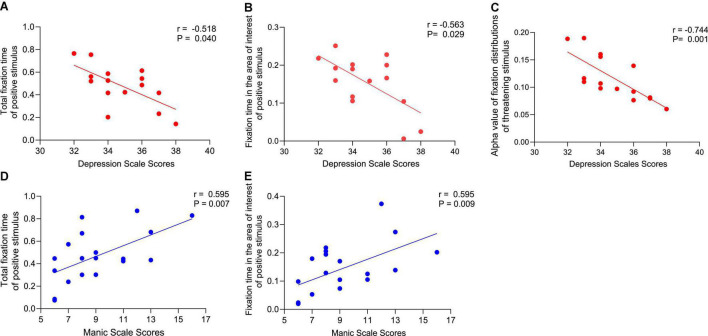
Correlation analysis between scale scores (depression scale scores and manic scale scores) and eye-tracking measures [total fixation time, area of interest (AOI) fixation time, and alpha value of fixation distributions]. Correlations between depression scale scores and **(A)** total fixation time of positive stimulus, **(B)** fixation time in the area of interest of positive stimulus, **(C)** alpha value of fixation distributions of threatening stimulus. Correlations between manic scale scores and **(D)** total fixation time of positive stimulus, **(E)** fixation time in the area of interest of positive stimulus. The correlation coefficient (r) and significance value (p) are also shown in each case. All the correlations are statistically significant.

In the depressed group, analyses showed a significant correlation between depression scale scores and total fixation time while looking at the positive stimuli (*r* = −0.518, *P* = 0.040) but not the threatening stimuli (*r* = −0.006, *P* = 0.983), the neutral stimuli (*r* = −0.121, *P* = 0.656), or the dysphoric stimuli (*r* = −0.235, *P* = 0.380) (Figure 4A). We further found that depression scale scores were negatively correlated with AOI fixation time while looking at the positive stimuli (*r* = −0.563, *P* = 0.029) (Figure 4B), but not with the threatening stimuli (*r* = 0.191, *P* = 0.479), neutral stimuli (*r* = −0.094, *P* = 0.729) or dysphoric stimuli (*r* = 0.289, *P* = 0.277).

Analyses revealed depression scale scores were negatively correlated with the alpha value of fixation distribution while looking at threatening stimuli (*r* = −0.744, *P* = 0.001) (Figure 4C), but not with the neutral stimuli (*r* = −0.151, *P* = 0.577), positive stimuli (*r* = −0.108, *P* = 0.692) or dysphoric stimuli (*r* = −0.217, *P* = 0.419).

#### Associations Between Manic Scale Scores for Mania and Eye-Tracking Measures Among Participants With Mania

Analyses revealed a significant correlation between the mania scale scores and the total fixation time while looking at the positive stimuli (*r* = 0.595, *P* = 0.007) (Figure 4D), but not the threatening stimuli (*r* = 0.214, *P* = 0.379), neutral stimuli (*r* = 0.279, *P* = 0.248) or dysphoric stimuli (*r* = 0.191, *P* = 0.433). We further found that mania scale scores were positively correlated with AOI fixation time while looking at positive stimuli (*r* = 0.595, *P* = 0.009) (Figure 4E), but not with the threatening stimuli (*r* = 0.267, *P* = 0.270), neutral stimuli (*r* = 0.254, *P* = 0.293) or dysphoric stimuli (*r* = 0.283, *P* = 0.241).

No associations were found between the mania scale scores and the alpha value of fixation distribution while looking at the different stimuli among adolescents with mania.

#### Associations Between Scale Scores and N1 Brain Activity

**FIGURE 5:**
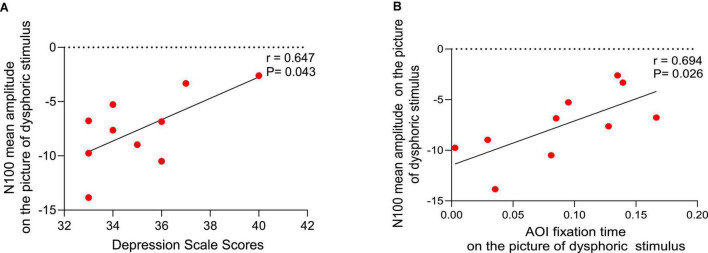
Correlation analysis. **(A)** Relationship between depression scale scores and the mean N1 amplitude while looking at the dysphoric stimuli. **(B)** Relationship between the mean N1 amplitude and area of interest (AOI) fixation time while looking at the dysphoric stimuli.

Analysis revealed that depression scale scores were significantly positively correlated with the mean N1 amplitude while looking at the dysphoric stimuli (*r* = 0.647, *P* = 0.043) (Figure 5A), but there were no significant correlations with the mean N1 amplitude while looking at the neutral stimuli (*r* = −0.202, *P* = 0.575), positive stimuli (*r* = −0.514, *P* = 0.193) or threatening stimuli (*r* = 1.05, *P* = 0.773).

However, no significant correlations were found between mania scale scores and the mean N1 amplitude while looking at the various stimuli for manic emotional tendencies.

#### Associations Between N1 Brain Activity and Eye-Tracking Measures

A positive correlation between the mean N10 amplitude and the AOI fixation time while looking at the dysphoric stimuli in the depressed group (*r* = 0.694, *P* = 0.026) (Figure 5B), but not the neutral stimuli (*r* = 0.003, *P* = 0.994), positive stimuli (*r* = 0.458, *P* = 0.215) or threatening stimuli (*r* = 0.417, *P* = 0.230).

For individuals with mania, we found no significant correlation between eye-tracking indices and the mean N1 amplitudes while viewing various stimuli.

## Discussion

In this study, we explored the group differences for eye-tracking characteristics, fixation dispersion degree, and ERP components with regard to different emotional visual stimuli in the free-viewing task for adolescents with emotional issues. Furthermore, we examined the association analysis between scale scores, eye-tracking measures, and ERP components across groups.

The eye-tracking results suggested no significant difference between the manic, depressed, and control groups in the total fixation time and AOI fixation time. However, there is a significant difference between the depressed group and the healthy control in fixation distribution, but no difference between the manic group and the healthy control. These findings suggest that total fixation duration in the free-viewing task did not reflect differences between depressive-prone and manic-prone adolescents, while the distribution of fixation was sensitive for the depression group. However, our study is inconsistent with the findings of Liu et al. (15) who found manic patients had a shorter total fixation duration on sad images and neutral images than healthy controls which reflected an avoidance of sad expressions, possibly due to inconsistent level of mania. Liu et al. studied manic patients with an average duration of episodes of 84 months, while we studied adolescents with manic tendencies screened by the scale. Individuals with depressive tendencies have more concentrated looking behavior with a smaller fixation dispersion than the control group, regardless of the type of emotional stimuli. These results are consistent with Eva Nouzová (38) studies that major depressive disorder (MDD) showed smaller fixation dispersion in the free-viewing eye movement task. In addition, Eva Nouzová also found that the smaller fixation dispersion was associated with lower verbal intelligence and verbal memory, which is a topic that we would like to address in the future.

Previous studies (39, 40) already showed that individuals with depressive tendencies reduced their fixation duration toward positive stimuli from different image sets than healthy controls. Our work further found a quantitative linear relationship in that higher depression scale scores were related to less total fixation time and less AOI fixation time toward positive but not threatening or dysphoric stimuli. According to existing theory, one possibility is that individuals with depressive tendencies do not have the motivation to maintain attention toward positive stimuli (40, 41). Ellis et al. (41) used Beck Depression Inventory-II (BDI-II) to assess symptoms of depression and designed an eye-tracking task with viewing a 2 × 2 array of emotional words. Results have revealed that individuals with BDI-II scores higher than 20 (which reached the threshold of depression) maintain shorter gazes with positive words relative to controls. Another possibility is that the mood-congruent attentional bias posited by cognitive patterns of depression raised by Beck (7) may have extended to the deficits toward positive affect (39, 40, 42). Shane et al. found that when participants were instructed to view an emotional picture (positive or negative) paired with a neutral image, depressed group showed less attention to positive stimuli than the healthy control group (42).

The ERP results confirmed that the N1 peak of individuals with depressive tendencies was significantly lower and slower than that of controls. We found that the activation of both groups was most pronounced in the parietal regions of the brain, and the individuals with depressive tendencies exhibited relatively longer latency and relatively lower arousal levels in the early stages of information processing than the healthy control group. This is consistent with previous studies (43, 44) that prolonged N1 latency may indicate a slower auto-arousal function in depression. Fotious et al. adopted pattern-reversed visual evoked potentials (PR-VEPs), which used checkerboard-flipped pictures as stimulus materials and recorded potential changes in the visual cortex for a sample of depression. They found depressed individuals were significantly longer N1 latency than non-depressed (44). According to (45), the decreased N1 amplitude may reflect the cognitive impairment of emotional stimuli processing in depression at the early stage. In literature (45), Jiu Chen adopted a visual emotional oddball paradigm in which participants needed to quickly point to a deviant face (happy or sad face) among the standard faces (neutral faces) by pressing a button. Results found depressed group had lower N170 amplitudes when identifying happy, neutral, and sad faces than healthy control. Therefore, these findings provide an excellent electrophysiological basis to prove that there exists processing bias toward emotional stimuli in depression in the early perceptual processing stage.

Based on the correlation between the N1 component and the DSM-V online self-assessment scales, depression scale scores were positively correlated with N1 amplitude toward dysphoric stimuli but not positive or threatening stimuli. This result may imply that individuals with higher depression scale scores allocated more cognitive resources toward dysphoric stimuli. According to (43), individuals with depressive tendencies are characterized by sensitivity to negative stimuli and impaired attention to positive stimuli at an early stage of the ERP signal. Therefore, individuals with depressive tendencies allocate more cognitive resources to process dysphoric stimuli, which would further affect their behavior and mood. In the long run, the vicious circle would seriously affect their physical and mental health (46).

The association between eye-tracking measures and ERP components in emotional issues has not yet been studied in the literature we read. Our study has yielded promising results that there were positive associations between AOI fixation time and N1 amplitudes toward dysphoric stimuli in individuals with depressive tendencies, but not positive or threatening stimuli. It indicates that the longer the AOI fixation time toward the dysphoric stimuli, the more cognitive resources were allocated. Researchers have shown that the attentional bias of adolescents with depressive tendencies is a product of a contemplative cognitive style in which they indulge in dysphoric content (47, 48). Also, individuals with depressive tendencies cannot get away from dysphoric content once they are focused, which is more pronounced with higher levels of rumination (47).

The ERP results confirmed no significant difference between the manic group and the control group in the N1 component. In contrast to the control group, these findings suggest that individuals with manic tendencies have relatively intact initial information processing. This is consistent with previous studies (28) that the N100 component in patients with manic or mixed bipolar disorder was studied, during an auditory discrimination task in which participants pressed keys to unusual 1,500 Hz tones interspersed in a series of 1,000 Hz tones. They reported that patients with bipolar disorder, which is also emotional issues, exhibited no reductions in N100 components compared to healthy controls. Our findings also confirmed that higher mania scale scores were associated with increased total fixation time or AOI fixation time toward positive stimuli. These results are consistent with previous studies (12) from Gruber et al. who adopted a dot-probe task to investigate attentional bias toward emotional faces and found manic proneness was positively associated with an attentional bias toward happy but not angry or fearful faces. However, our study is inconsistent with the findings of Rock et al. (49) who found no group difference in attentional bias toward positive stimuli among high-risk and low-risk undergraduate graduates with bipolar disorder, possibly because their study was not with emotional images stimuli, they employed the dot-probe task with emotional words and a different assessment for mania instead of DSM-V. In addition, no correlation between mania scale scores and N1 amplitude, or between N1 amplitude and eye-tracking output variables was found. Therefore, we have no conclusion about the association between their mania intensity and electrophysiological characteristics, and between visual behavior and electrophysiological characteristics.

Our study revealed the correlation between the participants’ attention and the persistence of emotional stimuli. Recent studies indicate that training anxious individuals diverted their attention from threatening stimuli, which reduced symptoms (50, 51). In addition, some studies suggest that the bias toward negative stimuli could be changed through training, which may have clinical effects (52). Therefore, our research can provide behavioral and electrophysiological targets for follow-up interventions to help adolescents with emotional issues recover.

We acknowledge several study limitations. First, the limitation of this study is the small sample size. However, it is worth noting that our findings did discover some potential associations which exist among eye-movement measures, ERP amplitudes and emotional scale scores despite the limitations associated with the current samples. Second, we assessed depressive and manic severity using DSM-V online self-assessment scales, with no formal clinical interviews to determine whether participants have reached the clinical diagnosis standard. Future studies should replicate this study in adolescents with depressive or manic tendencies and expand it to patients diagnosed with emotional disorders. Third, participants are limited to all-male adolescents, thus it is not clear whether gender differences have an effect on attentional bias. June Gruber et al. (12) found that when the participants were mainly females, emerging adults with hypomanic tendencies still had an attentional bias toward positive stimuli. It is consistent with our study that male adolescents with mania have an attentional bias toward positive stimuli. Fourth, due to no other scale data available (e.g., HAMD, HAMA, Young’s Mania Scale), the assessment of the severity of emotional issues was determined only based on DSM-V online self-assessment scales. Therefore, we will increase the ratings of other scales in future research.

## Conclusion

Our findings have shown that adolescents with depressive tendencies exhibit concentrated looking behavior, with reduced N1 amplitude and prolonged N1 latency, reflecting low and delayed arousal during the free-viewing task. The adolescents with depressive tendencies also showed impaired attention to positive stimuli and sensitivity to dysphoric stimuli. However, the adolescents’ behavior with manic tendencies coincides with heightened positive emotional responses. These findings provide preliminary support that attentional deviations, underlying neural mechanisms, and self-reported emotional issues occur at the same time, specifically helping to determine the interaction or potential causality among different emotional states, brain cognitive, and attentional bias. Future work is warranted in larger samples to continue to unpack the nature of cognitive processes. Thus, attentional processing and neural mechanisms may be of great significance to the maintenance and recovery from emotional disorders.

## Data Availability Statement

The raw data supporting the conclusions of this article will be made available by the authors, without undue reservation.

## Ethics Statement

The studies involving human participants were reviewed and approved by the Ethics Committee of the First People’s Hospital of Hefei. Written informed consent to participate in this study was provided by the participants or their legal guardian/next of kin.

## Author Contributions

BH conceptualized the study and research design. QW and XW designed the study, conducted the main data analysis, and drafted the manuscript. XW, RD, FZ, and SY collected the data. XW, RD, and SY managed the literature. All authors provided revisions to the final version and approved the submission.

## Conflict of Interest

The desktop eye-tracker system used in the experiment was jointly developed by our team and Dongguan Dongquan Intelligent Technology Co., Ltd. The coordinate output, sampling rate and accuracy of the eye tracker are no different from other self-developed and commercial eye trackers, which will not have any additional impact on the experimental results. The authors declare that the research was conducted in the absence of any commercial or financial relationships that could be construed as a potential conflict of interest.

## Publisher’s Note

All claims expressed in this article are solely those of the authors and do not necessarily represent those of their affiliated organizations, or those of the publisher, the editors and the reviewers. Any product that may be evaluated in this article, or claim that may be made by its manufacturer, is not guaranteed or endorsed by the publisher.
